# Short-term - change on physical capacities of football players within few days before ACL-injury: a retrospective case-control study

**DOI:** 10.1186/s13102-025-01518-3

**Published:** 2026-01-09

**Authors:** Andreas Kopf, Maximilian Getzreiter, Andreas Wittke, Emre Nokay, Markus Gesslein, Volker Alt, Werner Krutsch, Dominik Szymski

**Affiliations:** 1 Department of Orthopaedics and Traumatology, Paracelsus Medical University, Breslauer Strasse 201, Nuremberg, 90471 Germany; 2 Department of Sport Science, German University of Health & Sport, Ismaning, Germany; 3 Department of Trauma Surgery, University Medical Center Regensburg, Franz-Josef-Strauss-Allee 11, Regensburg, 93053 Germany; 4 FIFA Medical Centre of Excellence, University Medical Center Regensburg, Regensburg, Germany; 5 SportDocs Franken, Nuremberg, Germany

**Keywords:** Soccer, Prediction, Knee, Sports medicine, Performance, Football

## Abstract

**Purpose:**

Anterior cruciate ligament (ACL) ruptures represent one of the most severe injuries in professional football, often resulting in long rehabilitation, impaired performance, and increased risk of re-injury. The aim of this study was to investigate whether performance parameters derived from match statistics can serve as early indicators of ACL rupture in professional male football players.

**Methods:**

A retrospective case-control design was applied. Forty-two male professional football players from the German Bundesliga and 2. Bundesliga with confirmed ACL ruptures between 2016 and 2024 were included, alongside 42 matched controls from the same teams and positions. Match performance data from ten games preceding the injury were analyzed. Parameters included minutes played, total distance covered, number of sprints, maximal speed, pass accuracy, number of duels, and duel success rate. Independent t-tests compared injured and control players across individual matchdays and aggregated intervals (the average values across the last four, three, and two matches before injury). Additionally, odds ratios (OR) with 95% confidence intervals were computed based on upper (≥ 75th percentile) and lower (≤ 25th percentile) quartile thresholds to quantify the relative risk associated with extreme performance values.

**Results:**

Injured players showed higher maximum speed that consistently differentiated them from controls, with significant differences at matchday 2 (*p* = 0.005, OR = 3.42, 95% CI 1.45–8.06) and across all aggregated intervals (*p* = 0.015–0.031). Injured players also showed significantly fewer minutes played at matchday 2 before injury (*p* = 0.046, OR = 2.36, 95% CI 1.01–5.51) and across certain intervals (last four and three matches before injury; *p* = 0.027–0.044). Analysis of matchdays 5–10 revealed no significant group differences for any performance parameter, confirming that relevant performance changes manifest primarily in the immediate pre-injury period. No significant group differences emerged for distance covered, sprint count, pass accuracy, or duel frequency between ACL injured players and controls.

**Conclusion:**

Maximum speed showed the strongest association with ACL rupture risk, with significant differences at matchday 2 (*p* = 0.005) and across aggregated intervals (the average values across the last four, three, and two matches before injury). Reduced playing time emerged as an additional indicator. Although distance covered, sprint count, and pass accuracy did not reach statistical significance individually, their temporal patterns revealed a predisposing risk constellation: injured players demonstrated reduced cumulative exposure combined with acute high-intensity spikes at matchday 2, declining technical precision, and increased physical confrontation at matchday 1. This suggests ACL injury risk manifests through deterioration of integrated performance capacity under acute load fluctuations rather than isolated thresholds. Multifactorial approaches integrating biomechanical, physiological, and temporal performance patterns are essential for effective ACL injury prevention in professional football.

## Introduction

Anterior cruciate ligament (ACL) ruptures are among the most devastating injuries in professional football, imposing long rehabilitation times, impaired career trajectories, and considerable financial costs for clubs [1, 2]. Despite advances in surgical reconstruction, many players do not regain their pre-injury performance levels and may even face premature career termination [3, 4]. Moreover, the risk of re-injury remains substantial, with recurrence rates of up to 18% in German professional leagues [3, 5], and long-term sequelae such as post-traumatic osteoarthritis frequently occur [6, 7].

Given the high burden of ACL ruptures, preventive strategies are of critical importance. While several modifiable and non-modifiable risk factors have been identified—including neuromuscular control, fatigue, anatomical predispositions, and prior injury history [8, 9]—predicting ACL ruptures at the individual level remains challenging. With the widespread implementation of global positioning system (GPS) tracking and systematic match analysis, professional clubs now routinely monitor external and internal load parameters [10, 11]. These data not only guide training load management but may also provide early-warning signals for impending injuries [12]. This is particularly relevant as ACL injuries frequently occur as non-contact injuries, suggesting that neuromuscular deficits could potentially be detected beforehand as indicators of fatigue and increased injury risk [13, 14].

Despite the growing body of literature on injury prediction in football, a critical gap remains: no previous study has specifically investigated whether match-derived performance parameters can predict ACL rupture in professional players. Previous research has primarily focused on general injury risk and soft-tissue injuries, with studies specifically addressing ACL rupture prediction remaining scarce [15, 16]. This represents a significant limitation, as ACL ruptures differ fundamentally from muscle injuries in their etiology—they typically result from complex biomechanical events rather than cumulative load alone. Moreover, most investigations rely on training data rather than official match statistics, despite competitive matches imposing the highest physiological and mechanical demands on players [17, 18]. The present study therefore addresses this gap by being the first to systematically analyze match-specific performance patterns in the days and weeks immediately preceding ACL rupture, using data from professional German football leagues.

The novelty of this approach lies in three key aspects: First, we focus exclusively on ACL injuries rather than general injury risk, allowing for injury-specific pattern recognition. Second, we utilize official match statistics that reflect real competitive demands, rather than training load data. Third, we examine short-term performance changes in the immediate pre-injury period (up to ten matches), providing actionable insights for injury prevention strategies. By demonstrating that relevant performance alterations manifest primarily in the final four matches before rupture, this study offers practical guidance for targeted monitoring windows in professional football.

The present study therefore aimed to investigate whether performance parameters derived from official match statistics could predict ACL rupture in professional male football players. Specifically, we examined whether differences in playing time, distance covered, sprints, maximal speed, pass accuracy, duels, and duel success rate could be identified in the weeks preceding an ACL rupture compared to matched controls.

## Methods

### Study design

A retrospective, quantitative case-control study was conducted to investigate whether match-derived performance parameters can serve as predictors of anterior cruciate ligament (ACL) rupture in professional male football players. The study design was chosen because it allows for the comparison of injured players with matched controls using existing performance data, thereby enabling the identification of potential pre-injury performance patterns without the need for prospective longitudinal data collection being a feasibility study [19].

### Data sources

Two primary data sources were used. The German ACL register (*VKB-Register*), established in the 2014/15 season, this register systematically documents ACL ruptures across different levels of German football [13]. For this study, it provided verified cases of ACL rupture among players from the Bundesliga and 2. Bundesliga (first and second division). The Kicker.de database, a sports media platform, offers detailed match statistics for Bundesliga players, including official match appearances, playing time, and key performance indicators. It was used to extract match performance data for the ten games preceding each ACL rupture for injured player and their control.

### Participants

The ACL group consisted of professional male football players who sustained an ACL rupture between the 2016/17 and 2023/24 seasons while actively competing in the first or second Bundesliga. Inclusion criteria were: (I) confirmed ACL rupture listed in the *ACL-Register*, (II) active contract in the Bundesliga or 2. Bundesliga at the time of injury, and (III) availability of performance data from at least four official matches prior to the injury. Exclusion criteria included goalkeepers (due to distinct movement and injury profiles), players with incomplete match data (e.g., long-term absence before injury), or those with inconsistent team membership in the observation window.

Controls were selected based on strict matching criteria to reduce confounding. Each injured player was paired with one non-injured control from the same club, playing in the same or a mirrored position (e.g., left vs. right full-back). Controls had to feature in a similar number of matches within the same ten-match window. Players with ACL rupture during the observation period were excluded from the control pool.

After applying inclusion and exclusion criteria, the final dataset included 42 injured players and 42 matched controls, resulting in 84 professional male football players analyzed.

### Variables and performance parameters

The following variables were extracted. Anthropometric and demographic data included age, height, and weight for baseline homogeneity. Performance parameters comprised minutes played per match, total distance covered in kilometres, number of sprints as absolute count per match, maximum speed in km/h, pass accuracy as percentage, number of duels per match, and duel success rate as percentage. These variables were selected based on prior evidence linking them to injury risk, external load monitoring practices, and data availability in *Kicker.de* [10, 12].

### Observation period

For each injured player, data from the ten official matches immediately preceding the ACL rupture were analyzed. If the injury occurred during a match, that game was included as *matchday 0*. In case of training injuries, the most recent competitive match prior to the event defined *matchday 0*. Consequently, the observation window covered matchdays − 10 to 0.

To ensure adequate data coverage, a secondary analysis was also conducted using the final four matches before injury, as not all players had complete ten-match datasets. This allowed for comparison across three time intervals: the last four matches before rupture, the last three matches before rupture, and the last two matches before rupture. These aggregated intervals represent the average values of the respective parameters across the specified matchdays.

### Data preparation

All performance data were manually collected from *Kicker.de* and entered into a structured spreadsheet. Injured players and their controls were paired side-by-side to facilitate direct comparison. Where data were missing due to player rotation, suspension, or minor injuries, cases with fewer than four complete match datasets were excluded.

### Statistical analysis

Data analysis proceeded in two steps. Descriptive statistics were calculated (mean ± standard deviation) for all variables at each matchday and across aggregated intervals. Inferential statistics: Independent t-tests compared injured players with controls. Statistical significance was defined as *p* < 0.05. Additionally, odds ratios (OR) with 95% confidence intervals were computed based on upper (≥ 75th percentile) and lower (≤ 25th percentile) quartile thresholds to quantify the relative risk associated with extreme performance values. Analyses were conducted using Microsoft Excel (Microsoft Corp., Redmond, WA, USA) and SPSS Statistics version 28.0 (IBM Corp., Armonk, NY, USA).

## Results

### Descriptive characteristics

The final sample comprised 84 professional male football players (42 ACL rupture cases, 42 matched controls). Mean age at injury was 25.7 ± 3.2 years in the ACL group and 25.4 ± 3.6 years in the control group. No significant differences were observed in age, body height, or body weight between groups, confirming homogeneity of anthropometric characteristics. Twenty-eight (66.7%) of the ACL injuries occurred during official matches, while fourteen (33.3%) were sustained during training.

### Match performance parameters

Table 1 presents the comprehensive match performance data for all parameters across matchdays 0–10, including statistical comparisons between the ACL group and controls. The sample size (n) varies across matchdays because not all players were selected for every match due to squad rotation, tactical decisions, suspensions, or minor injuries that did not require exclusion from the study.

**Table 1:** Match performance parameters across matchdays before ACL injury

Matchday	10	9	8	7	6	5	4	3	2	1	0
Minutes played (Injured, *n* = 42)	69.7 ± 30.4 (*n* = 30)	69.4 ± 28.8 (*n* = 28)	69.0 ± 31.3 (*n* = 32)	71.1 ± 28.3 (*n* = 32)	74.2 ± 22.8 (*n* = 34)	71.1 ± 29.5 (*n* = 39)	72.3 ± 27.3 (*n* = 40)	70.2 ± 28.1 (*n* = 39)	76.0 ± 23.7 (*n* = 34)	74.7 ± 23.6 (*n* = 36)	36.5 ± 25.9 (*n* = 28)
Minutes played (Control, *n* = 42)	79.1 ± 19.5 (*n* = 25)	75.7 ± 27.8 (*n* = 25)	71.6 ± 30.1 (*n* = 29)	73.6 ± 25.1 (*n* = 31)	74.4 ± 24.8 (*n* = 36)	73.6 ± 23.2 (*n* = 34)	76.7 ± 23.3 (*n* = 38)	76.7 ± 25.6 (*n* = 40)	82.2 ± 21.4 (*n* = 39)	78.4 ± 20.6 (*n* = 39)	15.0 ± 0.0 (*n* = 1)
OR (95% KI)	-	-	-	-	-	-	-	-	2.36 (1.01–5.51)	-	-
p value	0.767	0.895	0.744	0.987	0.632	0.403	0.936	0.258	**0.046**	0.206	< 0.001
Distance covered in km (Injured, *n* = 42)	8.6 ± 3.6 (*n* = 27)	8.7 ± 3.2 (*n* = 25)	8.3 ± 3.8 (*n* = 30)	8.4 ± 3.5 (*n* = 30)	9.0 ± 2.1 (*n* = 33)	8.5 ± 3.5 (*n* = 31)	9.1 ± 2.8 (*n* = 36)	8.5 ± 3.3 (*n* = 39)	9.0 ± 2.8 (*n* = 29)	9.0 ± 2.9 (*n* = 32)	4.3 ± 3.3 (*n* = 27)
Distance covered in km (Control, *n* = 42)	9.6 ± 2.7 (*n* = 21)	9.2 ± 3.5 (*n* = 22)	8.7 ± 3.5 (*n* = 26)	9.0 ± 2.6 (*n* = 29)	9.0 ± 2.9 (*n* = 35)	8.9 ± 2.5 (*n* = 27)	9.4 ± 2.4 (*n* = 34)	9.1 ± 3.0 (*n* = 40)	9.5 ± 2.5 (*n* = 34)	9.5 ± 2.6 (*n* = 36)	0.8 ± 1.2 (*n* = 2)
OR (95% KI)	-	-	-	-	-	-	-	-	-	-	-
p value	0.293	0.658	0.679	0.414	0.994	0.620	0.714	0.369	0.430	0.513	0.150
Sprint count in n (Injured, *n* = 42)	15.3 ± 9.6 (*n* = 27)	17.1 ± 9.8 (*n* = 25)	16.2 ± 9.5 (*n* = 30)	16.0 ± 10.6 (*n* = 30)	18.0 ± 9.5 (*n* = 33)	16.1 ± 9.3 (*n* = 31)	16.9 ± 7.4 (*n* = 36)	14.0 ± 6.9 (*n* = 39)	18.7 ± 8.2 (*n* = 29)	15.9 ± 8.2 (*n* = 32)	8.9 ± 8.2 (*n* = 27)
Sprint count in n (Control, *n* = 42)	17.7 ± 6.6 (*n* = 22)	15.2 ± 7.4 (*n* = 22)	15.5 ± 8.3 (*n* = 27)	15.9 ± 6.8 (*n* = 29)	17.0 ± 9.0 (*n* = 35)	17.6 ± 8.9 (*n* = 27)	16.4 ± 9.0 (*n* = 34)	18.8 ± 7.8 (*n* = 40)	16.6 ± 7.1 (*n* = 34)	17.0 ± 9.5 (*n* = 36)	4.0 ± 0.0 (*n* = 1)
OR (95% KI)	-	-	-	-	-	-	-	-	-	-	-
p value	0.442	0.463	0.764	0.965	0.657	0.459	0.748	0.101	0.314	0.616	0.564
Maximum speed in (km/h) (Injured, *n* = 42)	30.6 ± 2.2 (*n* = 27)	30.7 ± 2.0 (*n* = 25)	30.4 ± 2.7 (*n* = 30)	30.1 ± 3.1 (*n* = 30)	30.7 ± 1.7 (*n* = 33)	30.5 ± 2.0 (*n* = 31)	31.2 ± 1.5 (*n* = 36)	31.1 ± 1.6 (*n* = 39)	31.5 ± 1.7 (*n* = 29)	31.1 ± 91.5 (*n* = 32)	30.2 ± 1.4 (*n* = 27)
Maximum speed in (km/h) (Control, *n* = 42)	30.3 ± 1.7 (*n* = 21)	30.0 ± 2.0 (*n* = 22)	29.8 ± 2.0 (*n* = 26)	30.3 ± 1.2 (*n* = 29)	30.5 ± 1.6 (*n* = 35)	30.9 ± 1.5 (*n* = 27)	30.5 ± 1.7 (*n* = 34)	30.6 ± 2.0 (*n* = 40)	30.3 ± 1.5 (*n* = 34)	30.5 ± 1.8 (*n* = 36)	29.8 ± 0.0 (*n* = 1)
OR (95% KI)	-	-	-	-	-	-	-	-	3.42 (1.45–8.06)	-	-
p value	0.550	0.240	0.386	0.751	0.583	0.380	0.052	0.175	**0.005**	0.138	0.797
Pass accuracy in % (Injured, *n* = 42)	75.5 ± 17.2 (*n* = 29)	80.6 ± 13.1 (*n* = 28)	73.6 ± 17.6 (*n* = 30)	78.6 ± 14.8 (*n* = 31)	78.3 ± 14.9 (*n* = 34)	79.3 ± 14.0 (*n* = 39)	76.3 ± 14.7 (*n* = 40)	77.5 ± 17.3 (*n* = 39)	75.9 ± 15.4 (*n* = 34)	71.1 ± 19.7 (*n* = 36)	73.6 ± 26.9 (*n* = 28)
Pass accuracy in % (Control, *n* = 42)	76.9 ± 18.3 (*n* = 25)	83.0 ± 10.5 (*n* = 24)	79.3 ± 13.7 (*n* = 29)	80.0 ± 12.1 (*n* = 31)	74.9 ± 17.3 (*n* = 36)	79.6 ± 11.3 (*n* = 34)	79.8 ± 12.4 (*n* = 38)	79.2 ± 12.9 (*n* = 39)	79.4 ± 12.2 (*n* = 39)	75.1 ± 12.1 (*n* = 38)	100.0 ± 0.0 (*n* = 1)
OR (95% KI)	-	-	-	-	-	-	-	-	-	-	-
p value	0.730	0.503	0.168	0.681	0.381	0.925	0.266	0.630	0.279	0.290	0.344
Duels in n (Injured, *n* = 42)	8.9 ± 5.1 (*n* = 30)	8.1 ± 4.0 (*n* = 28)	9.0 ± 5.4 (*n* = 32)	8.6 ± 5.6 (*n* = 32)	8.9 ± 5.0 (*n* = 34)	8.5 ± 5.2 (*n* = 39)	9.1 ± 6.3 (*n* = 40)	8.5 ± 6.0 (*n* = 39)	8.9 ± 4.9 (*n* = 34)	9.7 ± 5.1 (*n* = 36)	4.8 ± 4.8 (*n* = 28)
Duels in n (Control, *n* = 42)	10.5 ± 4.5 (*n* = 25)	8.0 ± 5.2 (*n* = 25)	7.4 ± 3.8 (*n* = 29)	8.9 ± 5.6 (*n* = 31)	8.8 ± 4.4 (*n* = 36)	8.4 ± 4.3 (*n* = 34)	9.0 ± 4.7 (*n* = 38)	8.8 ± 4.5 (*n* = 40)	10.1 ± 5.4 (*n* = 39)	8.3 ± 3.9 (*n* = 39)	1.0 ± 0.0 (*n* = 1)
OR (95% KI)	-	-	-	-	-	-	-	-	-	-	-
p value	0.240	0.958	0.199	0.810	0.904	0.946	0.904	0.826	0.366	0.173	0.443
Duel success rate in % (Injured, *n* = 42)	51.8 ± 24.9 (*n* = 30)	54.6 ± 21.9 (*n* = 27)	53.1 ± 25.2 (*n* = 32)	48.3 ± 25.0 (*n* = 31)	56.6 ± 20.7 (*n* = 34)	54.6 ± 22.2 (*n* = 38)	52.3 ± 22.4 (*n* = 40)	52.8 ± 21.7 (*n* = 39)	52.7 ± 20.7 (*n* = 34)	52.4 ± 22.0 (*n* = 35)	50.0 ± 30.4 (*n* = 25)
Duel success rate in % (Control, *n* = 42)	57.8 ± 17.1 (*n* = 25)	48.4 ± 25.9 (*n* = 23)	50.6 ± 19.7 (*n* = 28)	53.5 ± 21.8 (*n* = 31)	44.7 ± 25.7 (*n* = 36)	50.7 ± 21.3 (*n* = 34)	51.6 ± 20.7 (*n* = 38)	53.0 ± 21.3 (*n* = 40)	50.5 ± 18.7 (*n* = 39)	47.0 ± 18.3 (*n* = 38)	0.0 ± 0.0 (*n* = 1)
OR (95% KI)	-	-	-	-	2.18 (1.04–4.55)	-	-	-	-	-	-
p value	0.312	0.366	0.676	0.383	**0.038**	0.450	0.875	0.962	0.624	0.258	0.119

Data presented as mean ± standard deviation. Bold *p* values indicate statistical significance (*p* < 0.05)

*OR* Odds Ratio, *CI* Confidence Interval

Sample size (n) varies across matchdays because not all players were selected for every match due to squad rotation, tactical decisions, suspensions, or minor injuries. Aggregated interval analyses (last four, three, and two matches) showed significant differences for minutes played (last four: *p* = 0.027, OR = 2.58, 95% CI 1.11–5.99; last three: *p* = 0.044, OR = 2.21, 95% CI 1.02–4.80) and maximum speed (last four: *p* = 0.031, OR = 2.87, 95% CI 1.10–7.46; last three: *p* = 0.026, OR = 3.04, 95% CI 1.18–7.82; last two: *p* = 0.015, OR = 3.65, 95% CI 1.30-10.23)

### Minutes played

**Fig. 1:**
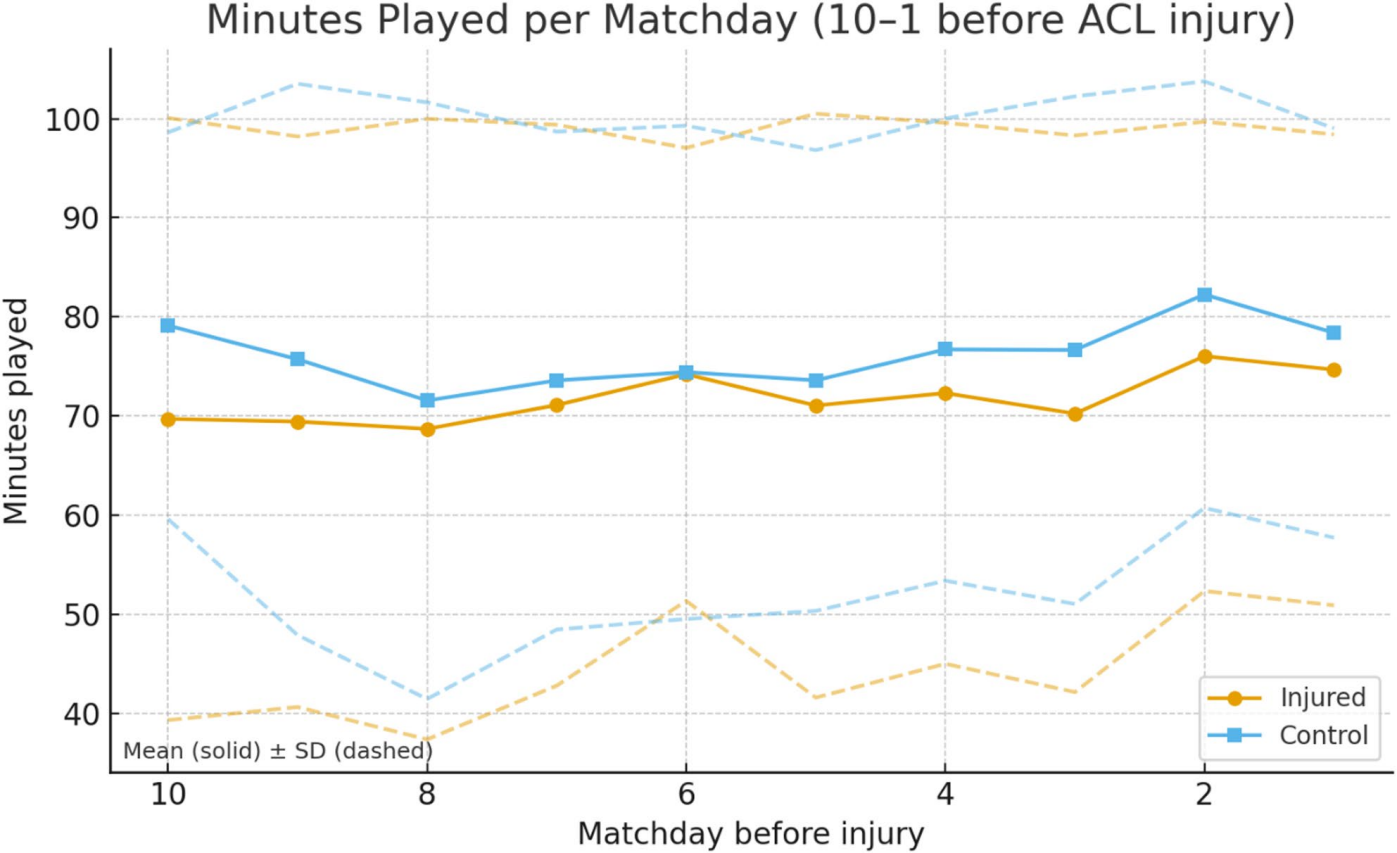
Played minutes per matchday before ACL injury for injured players and their uninjured control (Solid line being the mean value and dashed line being the standard deviation)

Across the ten matches prior to injury, injured players tended to accumulate fewer playing minutes compared to their controls. This difference became statistically significant at matchday 2 (*p* = 0.046, OR = 2.36, 95% CI 1.01–5.51), where injured players averaged markedly reduced exposure. Similar findings were observed when matchdays were aggregated: across the last four matches (*p* = 0.027, OR = 2.58, 95% CI 1.11–5.99) and last three matches (*p* = 0.044, OR = 2.21, 95% CI 1.02–4.80), injured players consistently recorded fewer minutes. Within the injured group, match exposure increased significantly from matchday 3 to 2 (*p* = 0.037), followed by a significant reduction from matchday 2 to 1 (*p* = 0.018) (Fig. 1).

### Distance covered

No significant differences in total distance covered were observed between injured players and controls across any single matchday or aggregated intervals. Both groups displayed comparable running volumes, averaging between 8.3 and 9.6 km per match (Table 1).

### Sprint count

The number of sprints per match did not significantly differ between groups across the ten-match observation window. Injured players tended to perform slightly more sprints in the final matches before injury, but the differences did not reach statistical significance (Table 1).

### Maximum speed

**Fig. 2:**
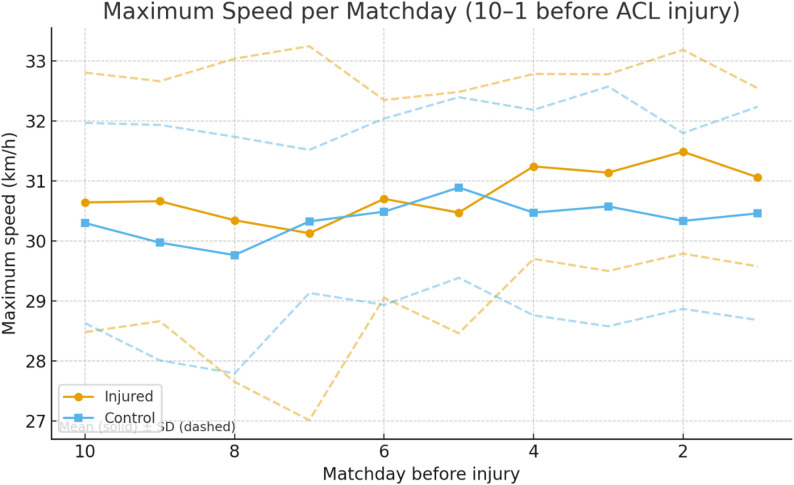
Maximum speed per matchday before ACL injury for injured players and their uninjured control (Solid line being the mean value and dashed line being the standard deviation)

Injured players demonstrated significantly higher maximum speeds than controls, particularly in the final matches preceding injury. At matchday 2, maximum speed was significantly greater in the **ACL** group (*p* = 0.005, OR = 3.42, 95% CI 1.45–8.06). Aggregated analysis confirmed this pattern: last four matches (*p* = 0.031, OR = 2.87, 95% CI 1.10–7.46), last three matches (*p* = 0.026, OR = 3.04, 95% CI 1.18–7.82), and last two matches (*p* = 0.015, OR = 3.65, 95% CI 1.30–10.23) all showed higher values in injured players (Fig. 2).

### Pass accuracy

Pass accuracy remained relatively stable in both groups throughout the observation period, averaging around 75–80%. No statistically significant differences were found at any matchday or interval (Table 1).

### Duels

The number of duels per match showed no consistent differences between groups. Injured players displayed slightly higher duel involvement at certain matchdays, but these trends did not achieve significance (Table 1).

### Duel success rate

**Fig. 3:**
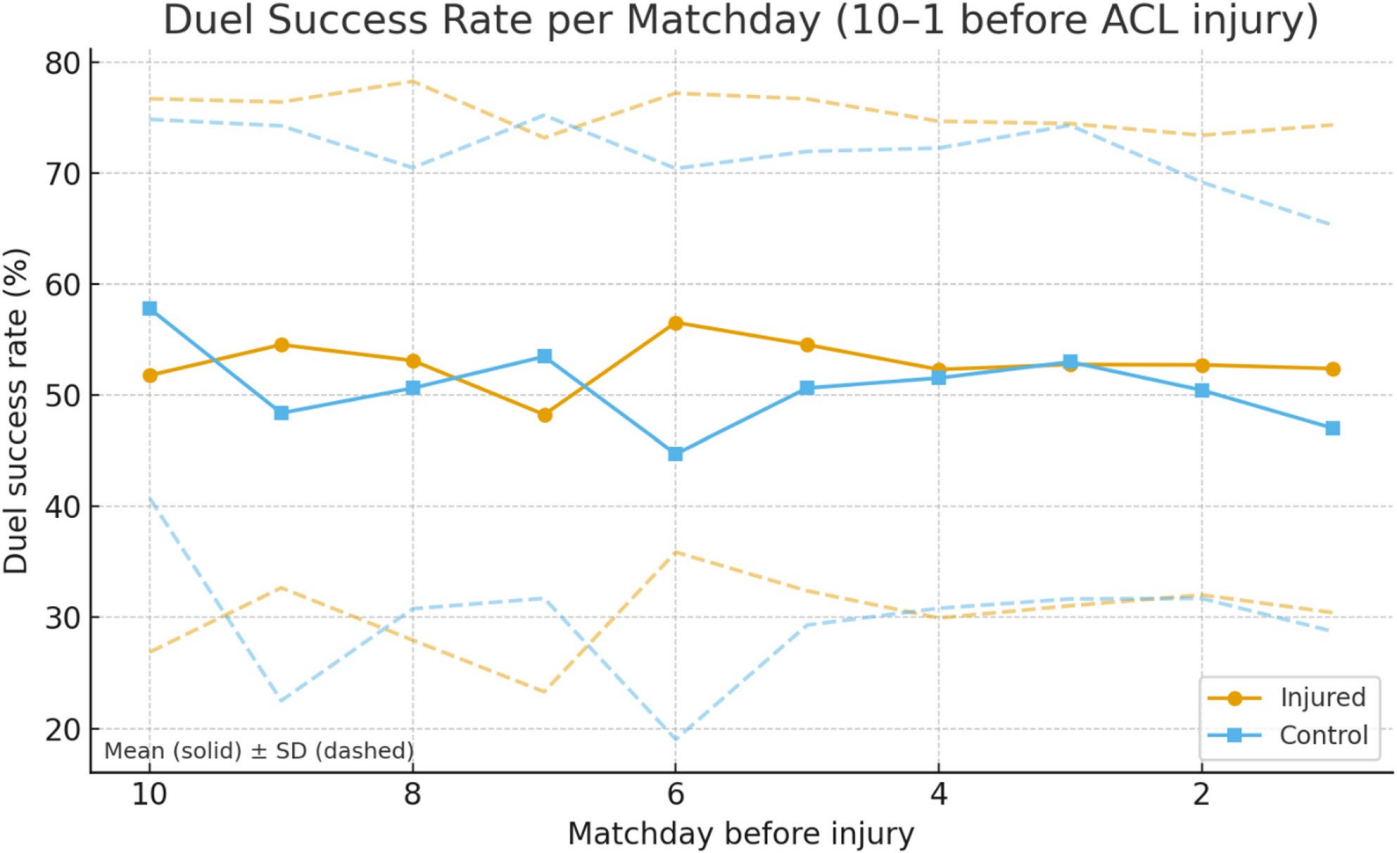
Duel success rate per matchday before ACL injury for injured players and their uninjured control (Solid line being the mean value and dashed line being the standard deviation)

At matchday 6, injured players exhibited a significantly higher duel success rate compared to controls (*p* = 0.038, OR = 2.18, 95% CI 1.04–4.55). Across other matchdays and aggregated intervals, no consistent group differences were observed (Fig. 3).

## Discussion

This retrospective case-control study investigated whether match-derived performance parameters could predict anterior cruciate ligament (ACL) rupture in professional male football players. The main findings were that (I) maximum speed consistently differentiated injured players from controls, (II) minutes played were significantly reduced in injured players in the final matches before rupture, and (III) duel success rate was significantly higher at one observation point. Other parameters, including distance covered, sprint count, pass accuracy, and duel frequency, showed no significant associations.

The most consistent and clinically relevant finding was that injured players demonstrated significantly higher maximum speeds in the matches preceding ACL rupture. High sprint velocities are known to impose substantial mechanical stress on the knee joint, particularly during deceleration, cutting, and landing movements that increase anterior tibial translation and strain on the ACL [20, 21]. In professional football, high-speed running and repeated sprint ability are decisive for performance [12], but these actions also represent critical risk scenarios. Our findings align with previous work showing that external load variables related to sprinting and accelerations are associated with greater injury incidence [16]. Importantly, this study extends prior research by demonstrating this link specifically for ACL ruptures, which have been underrepresented in earlier load-monitoring studies [15].

The analysis of the extended ten-match observation window provided important insights into the temporal specificity of performance changes. Notably, matchdays 5–10 revealed no significant group differences for any performance parameter, confirming that relevant performance alterations manifesting as injury risk signals concentrate in the immediate pre-injury period. This finding validates the clinical intuition that acute, short-term changes rather than distant historical performance patterns are predictive of imminent ACL rupture. The lack of significance in the earlier matchdays strengthens our conclusion that injury risk materializes through recent biomechanical and physiological deterioration rather than long-standing deficits. This temporal pattern has practical implications for monitoring strategies, suggesting that surveillance windows should prioritize the most recent 2–4 matches rather than extended historical data.

Another important observation was the acute workload pattern in the final matches before rupture. Injured players demonstrated a spike in high-intensity metrics (maximum speed, sprint count) two matches before injury, followed by a relative reduction in the immediate pre-injury match. This pattern must be understood within the broader context of cumulative load and integrated performance capacity. The critical insight is not simply that injured players experienced acute load spikes, but rather that these spikes occurred against a background of reduced overall match exposure. Specifically, injured players consistently covered less total distance across all matches compared to controls, indicating lower cumulative physical conditioning. When acute high-intensity demands (elevated maximum speed and sprint count at matchday 2) were superimposed on this foundation of reduced overall fitness, the result was a compromised ability to maintain neuromuscular control and technical precision. This manifested as declining pass accuracy at matchday 1 (71.1 ± 19.7% compared to 75–80% in earlier matches) and increased duel involvement (9.7 ± 5.1 duels at matchday 1), suggesting players were forced into more contested situations due to impaired ball control. The subsequent injury occurred not because of high absolute load, but because the acute intensity spike overwhelmed an already compromised physiological state characterized by insufficient cumulative conditioning. This load-capacity mismatch, where acute demands exceed the player’s current adaptive capacity built through recent match exposure, represents the mechanistic pathway through which ACL injury risk materializes. This pattern aligns with established theory, where sudden increases in load followed by incomplete recovery may compromise neuromuscular control and increase injury susceptibility [22]. In this context, reduced minutes could paradoxically indicate an elevated vulnerability to injury, either due to insufficient match rhythm or because coaches anticipated risk and attempted to mitigate it. Furthermore, the cumulative analysis revealed that injured players had lower overall match exposure across the observation period yet still sustained ACL ruptures.

A deeper examination of the non-significant parameters revealed meaningful patterns when considered holistically rather than in isolation. Although distance covered, sprint count, and pass accuracy did not individually differentiate injured from control players, their temporal trajectories within the injured group suggest a progressive deterioration in performance quality. Specifically, injured players demonstrated their poorest pass accuracy across the entire ten-match observation period at matchday 1 (71.1 ± 19.7%), while simultaneously engaging in their highest number of duels (9.7 ± 5.1 per match). This temporal coincidence suggests a mechanistic link: declining technical precision under fatigue may necessitate increased physical challenges, as players are forced into contested situations to compensate for imperfect ball control. Similarly, sprint count peaked at matchday 2 (18.7 ± 8.2) before declining at matchday 1 (15.9 ± 8.2), mirroring the spike-and-recovery pattern observed in maximum speed and playing time. Moreover, cumulative distance analysis revealed that injured players consistently covered less ground across all ten matches compared to controls, indicating lower overall match exposure yet paradoxically sustaining ACL ruptures. This apparent contradiction reinforces a critical insight: injury risk appears more closely tied to acute load fluctuations and movement quality deterioration than to absolute workload volume. The constellation of reduced cumulative load, acute high-intensity spikes, technical decline, and increased physical confrontation creates a biomechanical risk profile characterized by compromised neuromuscular control—precisely the conditions under which non-contact ACL injuries occur [23, 24]. These interconnected patterns underscore that single-parameter monitoring may miss critical warning signs that become apparent only through integrated performance profiling.

Contrary to expectations, total distance covered, sprint count, pass accuracy, and duel frequency were not significantly different between groups. These findings diverge from studies highlighting accumulated load and running volumes as risk factors [10, 17]. Several explanations are possible. First, unlike muscle injuries which demonstrate clear dose-response relationships with cumulative load, ACL ruptures often result from complex biomechanical events (e.g., valgus collapse, rotational forces) that may occur regardless of total volume. What matters is not simply how much a player runs, but rather the quality of neuromuscular control during high-risk movements. Second, the pattern observed in our data suggests that injury risk emerges from the interaction between cumulative conditioning status and acute load spikes rather than from either factor in isolation. Players with lower overall match exposure (reflected in reduced total distance) may lack the physical conditioning base necessary to safely tolerate acute high-intensity demands. When such players experience sudden increases in maximum speed or sprint count, their insufficient adaptive capacity renders them vulnerable to injury despite relatively modest absolute workloads. This explains why injured players covered less total distance yet still ruptured their ACL—the injury occurred not because of high load, but because acute demands exceeded their reduced capacity. Additionally, performance metrics such as distance covered may not adequately capture critical risk situations such as high-load decelerations, knee valgus moments, or awkward landings [24]. At last, technical-tactical demands, opponent pressure, and playing surface conditions are factors not represented in match statistics but may have contributed to injury occurrence.

Our findings confirm and extend previous research. Similar to Martins et al. (2023), we observed that sprint-related load was linked with injury risk, though their study primarily addressed muscular injuries. Alcantarilla-Pedrosa et al. (2021) reported associations between high-intensity running and ligament injuries in specific positions, which supports our observation of maximum speed as a critical factor. Conversely, Akenhead & Nassis (2016) concluded that performance monitoring alone may not suffice for injury prediction, a position consistent with our results showing limited predictive value of most parameters.

### Practical implications

The present findings have several implications for applied practice. Clubs should closely monitor peak velocity and performance values in the weeks preceding competition. Significant increases may warrant targeted load adjustments or preventive interventions. Decreases in match exposure should not automatically be seen as protective but may signal elevated injury vulnerability, particularly if combined with high-intensity exposures. Although preliminary, duel involvement and success may offer additional insights into mechanical load during match play.

Overall, the study suggests that performance data can provide early-warning signs but should be interpreted in the context of broader injury risk factors, including biomechanics, neuromuscular control, and recovery status.

### Limitations

Several limitations must be acknowledged. First, the study relied on secondary data sources, limiting the precision of performance metrics compared to GPS or wearable-based tracking systems. Second, training load - an essential component of total player exposure - was not available, which restricts interpretation to match-derived data. Third, the moderate sample size, though reasonable given the rarity of ACL ruptures, may have limited statistical power. Fourth, potential confounders such as prior knee injuries, limb alignment, and neuromuscular imbalances could not be controlled. Finally, data were restricted to German male professional football, and generalizability to other leagues or female athletes is uncertain.

## Conclusion

Match performance data offer valuable but limited insights into ACL rupture risk. Maximum speed demonstrated the strongest predictive potential, while playing time may also provide supplementary signals. However, the most instructive finding emerged from the holistic analysis of temporal performance patterns: the constellation of reduced cumulative match exposure, acute high-intensity spikes at matchday 2, declining pass accuracy, and increased duel involvement at matchday 1 collectively characterized the pre-injury profile. These interconnected patterns suggest that ACL injury risk manifests not through isolated parameter thresholds but through the deterioration of integrated performance capacity under acute load fluctuations. Single-parameter monitoring, while valuable, may systematically miss these warning signs. Performance parameters alone cannot fully capture the multifactorial nature of ACL injuries. Future research should integrate match and training load data, combine GPS-based kinematic information with biomechanical and neuromuscular screening, and apply advanced statistical or machine-learning models capable of detecting multivariate risk profiles rather than isolated predictors.

## Data Availability

Data available on request.
